# Drug-Death Related Bereavement and Social Support

**DOI:** 10.1177/00302228241238907

**Published:** 2024-03-13

**Authors:** Monika Alvestad Reime, Maja O’ Connor, Sigurd William Hystad, Kari Dyregrov

**Affiliations:** 1 Faculty of Health and Social Sciences, 1657 Western Norway University of Applied Sciences, Bergen, Norway; 2 Department of Psychology and Behavioural Sciences, 56606 Aarhus University, Aarhus, Denmark; 3 Department of Psychosocial Science, 1658 University of Bergen, Bergen, Norway

**Keywords:** drug-related death, bereavement, social support, stigma, withdrawal

## Abstract

The loss of a close one to drug-related death (DRD) has been characterized as a form of stigmatized bereavement, and research has shown that there is a high risk of bereavement complications. Social support can be a buffer against bereavement complications, but because of stigma, DRD bereaved persons access to social support can be challenged. Based on data from a Norwegian sample of DRD bereaved persons (*N* = 252) the present study examines (1) bereaved persons’ perceived access to different aspects of social support, and (2) the association between bereaved persons’ experiences of societal stigma, own withdrawal, self-blame, and their perceptions of social support. Results show (1) that bereaved persons’ access to contact with persons in the same situation is particularly low compared to other support aspects, and (2) that perceived stigma (4%) and own withdrawal (5%) predict variations in drug-related death bereaved persons’ perception of social support.

## Introduction

Drug-related death (DRD) is a major public health concern. In the USA the rapid increasing amount of DRDs have been referred to as an epidemic with 106,699 overdose deaths in 2021 (mortality rate of 34.3 per 100 000) (Centers for Disease Control and Prevention, 2023). In Norway, an average of 280 persons dies from overdose every year (mortality rate of 5.6 per 100 000) (Norwegian Institute of Public Health, 2023). Additionally, other death causes related to the illegal use of substances, such as deaths through accidents, violence, infectious disease, or other health disorders, also add to the high levels of DRD deaths. For every deceased person, there will be at least 10 close family members and friends left behind (Dyregrov et al., 2020).

The loss of a close person to a DRD has been shown to have a high risk of symptoms of complicated grief reactions and severe health implications (Bottomley et al., 2022; Christiansen et al., 2020; Titlestad & Dyregrov, 2022; Titlestad et al., 2022). Social support can be a buffer against the development of complicated grief reactions (Hibberd et al., 2010; Lobb et al., 2010), and in cases of sudden and violent losses, social support has been shown to act as a buffer against the development of PTSD (Scott et al., 2020). Furthermore, social support has been documented to be one of the most significant predictors of a healthy bereavement process (Burke & Neimeyer, 2013). However, when bereavement is stigmatized, access to support can be challenging, such as in the case of a DRD (Feigelman et al., 2012; Kalsås et al., 2023; Stout & Fleury-Steiner, 2023; Titlestad et al., 2021a).

Stigma can be described as the situation of an individual who is discredited and not fully accepted in society (Goffman, 2009). Stigma typically involves labelling, stereotyping, separation, status loss, and discrimination (Goffman, 2009; Link & Phelan, 2001). Stigma can be both societal and self-imposed (Link & Phelan, 2001), but in the case of a DRD stigma seems to be closely related to an association between the deceased’s way of life and the bereaved (Sheehan & Corrigan, 2020). Because of the stigma, guilt, and self-blame that often follows a DRD, persons left behind can be deprived of social support from their network, or they can withdraw and thereby make themselves unreachable by social support (Doka, 1999; Dyregrov et al., 2022; Dyregrov & Selseng, 2022; Valentine et al., 2016). In this article, we examine the association between societal stigma, withdrawal, self-blame, and social support for persons grieving a DRD.

Social support is a multifaceted construct (Wilsey & Shear, 2007), and the research field is characterized by a lack of consensus and clarity about how it best can be operationalized (Elklit et al., 2001; Scott et al., 2020; Vangelisti, 2009). However, an important division is made between peoples’ *perceptions* of the social support available at any time, and the actual support received in times of crisis (Joesph, 1999). Cobbs’ (1976, p. 300) definition of social support as “information leading the subject to believe that he is cared for and loved, esteemed, and a member of a network of mutual obligations” highlights the importance of peoples’ beliefs and perceptions of the support available rather than the quantity of support received. There is also evidence from research that perceived support can be as important as support received in the aftermath of a crisis, and there are indications that people’s perceptions of social support can be an important mediator for the usefulness of the support received (Dyregrov & Dyregrov, 2008; Wethington & Kessler, 1986). Social support can be divided into different types or aspects. Cohen and Wills (1985) present a definition of four types of support: informational, instrumental, self-esteem, and social companionship (the sense of belonging). Support can be given both by formal (professional) supporters and informal supporters. In this article, we focus on perceived informal support from family, friends, and peers (individuals with similar experiences supporting each other), as measured quantitatively.

Studies have actualized different variables that can affect peoples’ (perceived) access to support. A systematic review identified that being a woman is generally associated with receiving more positive support following bereavement than men (Logan et al., 2018). For example, men experienced fewer opportunities to talk about the DRD and their feelings around it and were perceived as having more difficulties in talking about grief and loss, particularly in later life (Logan et al., 2018). Furthermore, studies have discussed whether elderly persons have less access to social support because of, for example, physical health challenges and the reduced size of a network of people the same age, though no consistent conclusions have been made (Elklit & O’Connor, 2005; Wrosch et al., 2013). The time since death has also been discussed as a factor that can mediate access to social support. Often, the strongest support initiatives are found in the immediate period after a death, and then they gradually decrease as time goes by (Dyregrov, 2006; Titlestad et al., 2021b). Another variable that might have an influence on support, is the bereaved persons’ relation to the deceased, for example, how some losses can be more significant to some family members than others (Gilbert, 1996). The term hierarchy of grief has been used to describe the positioning of the bereaved as more or less legitimate mourners, typically placing parents and spouses at the top of the hierarchy (Peskin, 2019).

Studies on DRD bereaved persons and social support are scarce. A study from the same population as the present article (the END-project) explored the social health domain of persons bereaved by a DRD (*N* = 255) and found the mean score for social support to be relatively low compared with other bereaved populations (Kalsås et al., 2023). The study did not go further into the different aspects of support. Another qualitative study from the END-project on siblings’ bereavement found that informal support failed (Dyregrov et al., 2022). Barriers for experienced support identified were intrafamily phenomena such as difficult communication, complex family relations, and an difficult environment during their upbringing. However, societal phenomena such as stigma, a feeling of blame, and guilt also acted as a barrier to the siblings from seeking support outside of the family (Dyregrov et al., 2022).

Several studies have explored stigma relating to DRD and found DRD bereavement to be highly stigmatized (Bottomley et al., 2023; Dyregrov & Selseng, 2022; Feigelman et al., 2011, 2012; Stout, 2022; Stout & Fleury-Steiner, 2023). In a study from the END-project, Dyregrov and Selseng (2022) found that nearly half of a sample of bereaved family members and friends (*N* = 255) experienced derogatory comments from family, friends, neighbours, media, or professionals regarding the deceased. In a qualitative study from the USA, DRD bereaved participants described experiencing stigmatization in interactions with law enforcement, friends, and family. They felt excluded by friends, and isolated from their family, and they experienced being blamed for the death. Stigma also became a barrier for seeking support, for example from self-help groups (Stout & Fleury-Steiner, 2023).

Bottomley et al. (2023) compared levels of stigma, guilt, and shame among persons bereaved by death due to an overdose to persons bereaved by a suicide. They found no significant differences between the two samples, though an important take-away was that the levels of stigmatization, guilt, and shame were high in both groups. Another study compared attitudes towards opioid deaths and suicide by using an online survey where participants were randomized to either respond to a hypothetical case of opioid overdose (*N* = 229) or a case of suicide (*N* = 274). Results showed that respondents were significantly more likely to intervene in the suicide situation than in the opioid overdose situation (Kheibari et al., 2021). The study also showed that in the opioid death scenario, the participants were significantly more likely to characterize the deceased using words such as “pathetic”, “an embarrassment”, “irresponsible”, and “stupid”. In contrast, those who died by suicide were more likely to be described as “brave” and “dedicated” (Kheibari et al., 2021).

To our knowledge, no studies have examined DRD bereaved persons’ perceptions of informal social support and factors that can influence perceptions of social support in more detail. This study aims to contribute to filling in this knowledge gap through the following research questions: (1) In what ways do DRD bereaved persons perceive different aspects of social support, and how does the perceived social support vary with the bereaved persons’ gender, age, time since death and relation to the deceased? (2) In what ways does the total level of perceived social support vary with the bereaved persons’ gender, and reported level of experienced societal stigma, self-blame, and own withdrawal?

## Methods

The current study is based on quantitative data from a large project on DRD bereavement and recovery in Norway (the END-project). The project has a cross-sectional design, and the survey for the bereaved persons used in the current study was administered in the period between March and December 2018. This survey consists of several validated questionnaires, some of which have been used in the other quantitative studies from the END-project referred to in this article (e.g. Kalsås et al., 2023; Titlestad et al., 2022).

### Recruitment and Sample Characteristics

The recruitment of the sample of bereaved family members and close friends took place in 2018 in cooperation with municipalities in Norway, NGOs, and relevant support services, and through advertising (e.g., social media), and the “snowballing” method. Inclusion criteria were more than three months since the loss of a family member or close friend to a DRD (no restriction was set for an upper time limit), Norwegian speaking, and over 18 years old. In total, 255 bereaved family members and close friends completed the survey. Of these, 252 completed the questions on social support examined in this article. The sample (*N* = 252) consisted of 94 (38%) parents, 79 (31%) siblings, 51 (20%) other family members (children, grandparents, and other family members), and 28 (11%) non-family members (friends, partners, ex-partners). Eighty-one percent (*N* = 205) were females. The average time since death was *M* = 97 months (range 3–420), or 8.1 years, and the average age of respondents was *M* = 47 years (range 18–80). The education level in the sample was high, with 219 (87%) of the respondents having completed either senior high school or college/university. The average age of the deceased at time of death was *M* = 31 years. Most respondents were married, *N* = 160 (63%), and the respondents perceived their relation to the deceased at time of death to be close, *M* = 4.5 on a five-point Likert scale from 1 (not close) to 5 (very close). See Table 1.

**Table 1.** Sample Characteristics (*N* = 252) With Mean (M), Standard Deviance (SD), Range, and *N*%.

Variable	M	SD	Range	*N* (%)
Female sex				205 (81.3)
Relation to the deceased				
Parent				94 (37.3)
Sibling				79 (31.3)
Other family-member				51 (20.2)
No family-member (close friends, partners)				28 (11.1)
Experienced closenes**s** to the deceased (1–5) *1 = not close, 5 = very close*	4.5	.8	4	
Time since death (in months)	97.4	88.5	417	
Age of the bereaved at time of survey	47.4	13.9	62	
Age of the deceased at time of death	31.4	9.9	53	
Bereaved’ educational level				
Primary school				32 (12.7)
Senior high school				94 (37.3)
College/University				125 (49.6)
Marital status				
Married/cohabitant				160 (63.5)
Boyfriend/girlfriend				17 (6.7)
Widow/widower				8 (3.2)
Divorced/separated				25 (9.9)
Singel				41 (16.3)
Occupational status				
Full-time				106 (42.1)
Part-time				35 (13.9)
Seek leave				11 (4.4)
Retired				29 (11.5)
Student				14 (5.6)
Other				56 (22.2)

### Measurements

#### The Crisis Support Scale (CSS)

The CSS consists of seven items measuring social support (Elklit et al., 2001; Joseph et al., 1992). The five first items measure perceived positive support: (1) “Someone willing to listen”; (2) “Contact with persons in a similar situation”; (3) “Able to talk about thoughts and feelings”; (4) “Sympathy and support from others”; (5) “Practical support”. The sixth item measures negative response; (6) “Feeling let down”, and the seventh item measures: (7) “Overall satisfaction with support received” (Elklit et al., 2001). The items are measured on a seven-point Likert scale. High scores indicate high perceived social support, except for item 6 where a high score indicates negative experiences with support. The CSS scale has shown to have good internal consistency across multiple studies, both measured as a seven-item scale (with item (6) reversed) (Arnberg et al., 2012; Elklit & Kurdahl, 2013; Elklit et al., 2001), and on a six-item scale, leaving out item (7) (Elklit et al., 2001; Jind et al., 2010).

In the present study, and in line with Kalsås et al. (2023) and Wahlström et al. (2013), we decided to use the five first items on *positive* support for the subscale analysis. The minimum level of social support is a value of 5, and the maximum level is a value of 35. Five imputations were made for respondents missing one value on the items (1) – (5). Imputations were done by calculating the mean value for the respondent’s other scores. The Cronbach alpha (α) for the five-items scale used in the present study was acceptable: .779.

#### Societal Stigma, Own Withdrawal, and Self-Blame

A scale for measuring stigma was developed from the “The Special grief questions” (SGQ). SGQ consists of 16 single items measuring the special conditions following a DRD (and is not a scale with sum score or cut-off scores) (Dyregrov et al., 2020; Titlestad & Dyregrov, 2022). The SGQ questions were developed around aspects and complex emotions such as experiences of guilt, shame, anxiety/fear of death, anticipated grief, experiences of stigma, ambivalence (feeling relief/guilt for feeling relief) and items related to communication and disclosure of the cause of death. The items are rated on a five-point Likert scale from 1 (to a small extent) to 5 (to a great extent). From the SGQ, five items were chosen that describe respondents’ experiences of societal stigma: (1) “Others blame me for the death”; (2) “I experience that others expect me to be ashamed of the death”; (3) “Others are talking negatively behind my back because of him/her”; (4) “I have experienced stigmatizing comments in social media”; (5) “I experience that others do not think I have the right to grieve”. A sum score was made to measure the total perceived societal stigma (minimum 5, maximum 25). Correlation analyses showed that the five stigma items were all significantly related to one another (*p* < .0005). Cronbach’s alpha (α) for the total score on the five-item scale was acceptable: .795.

Self-blame was analysed as a single item from the SQG: “I blame myself for the death” and rated on a five-points Likert scale from 1 “to a small extent” to 5 “to a great extent. Own withdrawal was analysed as a single item from the Assistance Questionnaire (AQ) (Dyregrov et al., 2003): “I have withdrawn from others”. The question was measured on a five-point Likert scale, from 1 “not at all” to 5 “to a great extent”.

### Statistical Analyses

The mean scores were calculated for the overall positive support scale and the seven single items to measure how the bereaved perceived both their total positive support and the different aspects of support. One-way ANOVAs and independent samples *t*-tests were conducted to measure subgroup variations in both total perceived support and the single items.

Cohen’s d was used as an effect size measure in the *t*-tests, while eta squared (η2) was used for the ANOVA models. A general guideline for interpreting Cohen’s *d* is to refer to the effect as small (*d* = 0.2), medium (*d* = 0.5), and large (*d* = 0.8), as originally suggested by Cohen (1998). The corresponding guideline for η2 is to refer to η2 = .01 as small, η2 = .06 as medium, and η2 = .14 as large.

Bivariate correlations (Pearson’s product moment coefficient (*r*)) were used to measure the associations between social support (total and single items) and the age of the bereaved person and time since death.

Potential predictors of social support were further examined in a hierarchical multiple regression analysis. Participants’ gender and their self-reported closeness to the deceased at the time of death were included as control variables in the first step, while the focal variables societal stigma, self-blame, and own withdrawal were entered in the second step.

The coefficient of determination (*R*^2^) and squared semi-partial correlations (*sr*^2^) were used as effect size measures for the linear regression. Whereas *R*^2^ is a statistical measure that determines the proportion of variations in social support scores that can be explained by the independent variables in combination, *sr*^2^ can be used to assess the proportions of the total variations that can be uniquely explained by each independent variable.

### Ethics

The END-project was approved in February 2018 by the Norwegian Regional Ethical Committees for Medical and Health Research Ethics (2017/2486/REK vest). The study follows ethical standards from the Helsinki Declaration. Data is managed and stored according to the Norwegian General Data Protection Regulation, instructions of the Regional Ethical Committee, and internal regulations of the Western Norway University of Applied Sciences (HVL). Respondents were provided with written information about the study before filling out the survey and were informed of the possibility of contacting the project manager should any negative reactions arise in the aftermath of answering the survey.

## Results

### In What Ways do DRD Bereaved Persons Perceive Different Aspects of Social Support?

Results on mean scores for different aspects of social support and mean scores on total perceived positive social support are presented in Table 2.

**Table 2.** Perceived Social Support With *N* (%) and Mean (M).

	*N* (%)	Total score	Mean						
			Item 1	Item 2	Item 3	Item 4	Item 5	Item 6	Item 7
Whole sample	252	24.1	5.1	3.9	5.1	5.2	4.8	4.3	4.4
Female	205 (81%)	23.7	5.0	3.8	5.0	5.1	4.8	4.3	4.3
Males	47 (19%)	25.8	5.5	4.6	5.3	5.4	4.9	4.0	4.6
Parent	94 (37%)	24.3	5.1	3.8	5.2	5.4	4.9	3.8	4.9
Siblings	79 (31%)	24.2	5.2	3.7	5.1	5.2	5.0	4.2	4.2
Other family members	51 (21%)	24.0	5.2	4.1	4.9	5.0	4.8	4.5	4.1
Non-family members	28 (11%)	23.5	4.8	4.7	4.6	4.8	4.6	5.6	3.4

*Note*s. Items (1) “Someone willing to listen”; (2) “Contact with people in a similar situation”; (3) “Able to talk about thoughts and feelings”; (4) “Sympathy and support from others”; (5) “Practical support”; (6) “Feeling let down”; (7) “Overall satisfaction with support received”.

The results show a mean score on single items on the seven item CSS scale of *M* = 4.6 (range 1–7). The lowest score was on (2) “contact with other persons in the same situation”: *M* = 3.9. Regarding single items, females scored significantly lower than men on item (1) “Someone willing to listen” (p = .046, *d* = .33), and on item (2) “Contact with persons in a similar situation” (p = .011, *d* = .39). Both effect sizes can be characterized as small. For single items (3) - (7), there were no significant differences regarding respondents’ gender. The mean total score on perceived positive support was *M* = 24.1 (SD =*:* 6.58, range: 6–35). Females scored significantly lower than males on perceived positive social support (p = .032, *d* = .31).

There were no significant differences on single items (1) - (5) or on perceived positive social support (sum score 1–5) for bereaved persons with different relations to the deceased (parent, sibling, other family members, non-family members). For analysis of item (6) “Feeling let down”, non-family members reported a significantly higher score than parents, siblings, and other family members (*p* < .001). The effect size of *η*^2^ = .09 can be characterized as medium in magnitude. For analysis of item (7) “Overall satisfaction”, parents reported being significantly more satisfied with support than siblings (*p* = .043), other family members (*p* = .025), and non-family members (*p* < .001). The effect size calculated by using eta squared was medium (*η*^2^ = .08).

There was no correlation between the age of the bereaved person at the time of the survey and single items (1) - (5), and on perceived positive social support (sum score 1–5). However, higher age correlated by a small, yet significant amount (r = .20) with lower scores on item (6) (“Feeling let down”), and a small, yet significant amount (r = .21) on higher scores on item (7) (“Overall satisfaction”). There was no significant correlation between the time since death and total perceived social support and single items (1) - (7). See Table 3.

**Table 3.** Correlations (r) of Positive Social Support and Items (1) to (7) With Age and Time Since Death.

Variables	Age of bereaved	Time since death
Social support	.07	.05
Item 1	.06	.08
Item 2	.00	.08
Item 3	.10	.06
Item 4	.10	−.06
Item 5	.03	.00
Item 6	−0.20***	.08
Item 7	0.21***	−.11

*Note*s. Items (1) “Someone willing to listen”; (2) “Contact with people in a similar situation”; (3) “Able to talk about thoughts and feelings”; (4) “Sympathy and support from others”; (5) “Practical support”; (6) “Feeling let down”; (7) “Overall satisfaction with support received”.

*p < .05, **p < .01, ***p < .001.

### Stigma, Self-Blame, and Own Withdrawal as Predictors for Positive Social Support

The mean levels of societal stigma in our sample were *M* = 9.52 (*SD* = 4.93, range 5–25) for the total stigma score, *M* = 2.67 (SD = 1.43, range 1–5) for self-blame, and *M* = 2.87 (SD = 1.15, range 1–5) for own withdrawal.

The results from the multiple regression showed that gender and self-reported closeness to the deceased at time of death accounted for a statistically insignificant proportion of the variations in perceived positive social support (*R*^2^ = .017, *F*[2, 240] = 2.058, *p* = .13). Entering the three focal variables societal stigma, self-blame, and own withdrawal increased the explained variations by 12.6% (Δ*R*^2^ = .126, Δ*F*[3, 237] = 11.567, *p* < .001). As seen in Table 4, stigma (*p* < .001) and own withdrawal (*p* < .001) were statistically significant predictors, while self-blame was not (*p* = .398). Higher levels of societal stigma (*b* = −0.30) and own withdrawal (*b* = −1.30) were associated with lower levels of support. The experienced societal stigma accounted for about 4% (*sr*^2^ = .041) and own withdrawal about 5% (*sr*^2^ = .049) of the variance in perceived positive social support.

**Table 4.** Hierarchical Multiple Regression: Societal Stigma, Self-Blame, and Own Withdrawal as Predictors for Perceived Positive Social Support (CSS 1–5).

Model	*B*	95% CI		ß	*sr* ^2^	*R* ^2^
		Lower bound	Upper bound			
Step 1						.017
Gender	2.08	−0.50	4.21	.12	.02	
Perceived closeness	0.38	−0.60	1.40	.05	.00	
Step 2						.142**
Gender	1.65	−0.38	3.68	.10	.01	
Perceived closeness	0.25	−0.69	1.19	.03	.00	
Self-blame	−0.26	−0.87	0.35	−.06	.00	
Own Withdrawal	−1.30**	−2.00	−0.61	−.23	.05	
Societal stigma	−0.30**	−0.47	−0.12	−.22	.04	

*Note. sr*^2^ = semipartial correlation coefficient.

**p < .001.

## Discussion

How the bereaved persons assess their support can be influenced by their expectations and desires regarding the different aspects of support (Reevy & Maslach, 2001), and must be understood within a particular historical and cultural context. For example, a study on traumatic bereavement from the Sámi areas in Norway has pointed to how traditional Sami norms and values that highlight self-mastery, self-reliance, and autonomy, can serve as an obstacle to asking for help from a professional network. Only help from the closest or the extended family was accepted, and no strangers were let in (Dyregrov et al., 2014). The following discussion takes departure in perspectives on bereavement and social support developed within a Western research tradition and applied to a sample of DRD bereaved persons mainly from the Norwegian middle class.

### Aspects of Social Support and Subgroup Variations

Results from the present study show some variances in access to different aspects of social support when measured on a subgroup level. Older age of respondents is correlated with higher scores on overall satisfaction and with less negative experiences with social support (“feeling let down”). Despite the fact that the number of supportive partners often decreases with old age, research has shown that the quality of the remaining network can be good (Elklit & O'Connor, 2005; Wrosch et al., 2013). For example, theories of aging have questioned how (reduced) life expectancies can lead to more focus on personal well-being and contribute to increasing older persons’ attentiveness to what emotionally meaningful relations are (Carstensen et al., 1999). Furthermore, in the wake of the loss, the bereaved may have filtered out and dropped out of less meaningful relationships as they got older (Dyregrov & Dyregrov, 2008). Older people have also gained more life experiences, and often experienced multiple losses, both related to loss of physical functioning, loss of siblings, and friends, and as like, loss of economic independence and change of residence (O’Connor, 2010). Hence, it can be reasonable to assume that older people have more coping strategies available, that for example can be a recourse for seeking social support. However, research has also shown that for example the loss of a partner in older age can be an extremely stressful life experience, that among others has proven to predict PTSD and other bereavement complications (Elklit & O'Connor, 2005; Treml et al., 2022).

Some variance is also found regarding the relationship of the bereaved to the deceased. Parents report both having fewer negative experiences with social support and being more satisfied overall with the social support they have received than bereaved persons with no familial relation to the deceased. The results can be understood in light of studies that have discussed a generational order in the ranking of grief, in which parents and spouses are seen as the most highly-ranked mourners, followed by children and other family members (Peskin, 2019). The loss of a child has also been conceptualized as one of the most devastating life events a person can experience (Peskin, 2019), probably strengthening the assumption of parents as being in need of the most support and attention. Close friends on the other hand are commonly placed lower down on the grief hierarchy than family members (Johnsen & Dyregrov, 2016; Liu et al., 2019).

Regarding total perceived social support, the only variance on the subgroup level relates to respondents’ gender, where women report significantly lower scores on positive social support compared to men. At first sight, this result diverges from other studies that have documented consistent variations between men and women when it comes to giving or receiving support (Matud et al., 2003; Reevy & Maslach, 2001). Here they find women to be more likely to participate in social relationships, to verbally express their emotions, and to seek intimacy (Matud et al., 2003; Stroebe et al., 2001). Women are also more likely to seek social support in stressful situations (Taylor, 2011). On the other hand, men are less emotionally expressive and put more emphasis on autonomy and personal independence (Matud et al., 2003). However, it can be hypothesized that women in the present study as a result of the above-mentioned diversity between men and women might place higher expectations on and experience a greater need for social support, and hence they describe less satisfaction than men when they report on their experiences with social support.

### Lacking Contact With Persons in the Same Situation

The results show a mean value for scores on different aspects of social support (*M* = 4.6). The lowest level was for the item (2) measuring “contact with persons in the same situation”, *M* = 3.9 (see Table 2). How the bereaved persons reported on this item indicated whether they had access to peer support, a support type that has shown to be highly valued after many types of deaths (Barlow et al., 2010; Bartone et al., 2019; Titlestad et al., 2020). Peer support involves some key principles of respect, shared responsibility, and mutual agreement on what is helpful (Dyregrov & Dyregrov, 2008). The END-study has documented a desire for peer support from both siblings and parents (Dyregrov et al., 2022; Titlestad et al., 2020). Specifically, the bereaved persons call for support groups that are exclusively for drug-related deaths. Sharing this unique experience can make it easier to understand each other, and easier to know how to talk about the loss (Titlestad et al., 2020). Access to peer support opens up opportunities to share common thoughts and experiences, and to disclose feelings that can be difficult to talk about with others (Dyregrov & Dyregrov, 2008). As such, it can be particularly important in the event of a stigmatized death (Bartone et al., 2019; Feigelman et al., 2020). Feigelman et al. (2020) have also found that helping others in a support group can be important in drug-death bereaved parents’ meaning-making process.

Taboos and stigma related to the death and its causes, can impede peer support. Results from the END-project show how the bereaved person can hide the cause of the death from their network, or that they avoid open communication about the deceased and their grief (Dyregrov et al., 2022). Their network can find it difficult to address deaths that are perceived as stigmatized, for example, DRD bereaved persons in the END-project described members of their support network who were unsure about what to say (Titlestad et al., 2020). The individual shame and self-stigma together with the societal stigma related to DRD bereavement may dominate the lives of the bereaved to such a degree that it may prevent them from gathering and benefitting from mutual support. Stigma and taboos surrounding the cause of death can also make it more difficult for peers to identify bereaved persons who share common experiences.

### Societal Stigma and Own Withdrawal are Associated With Less Access to Social Support

The results of the present study show that the three factors: societal stigma, own withdrawal, and self-blame, predict 12.5% of variations of perceived social support (when controlled for gender, and perceived closeness to the deceased). This result indicates that societal stigma can be associated with bereaved persons’ access to social support, or their effort to seek such support and that the unique contribution of societal stigma (4%) on social support is about the same size as the unique contribution of own withdrawal (5%).

Lack of social support and own withdrawal are closely connected, but it can be difficult to determine what is cause and what is effect (Dyregrov & Dyregrov, 2008). Self-isolation due to exhaustion and grief can make the bereaved persons inaccessible to their social network, but bereaved persons who experience stigma and less positive social support will also be more likely to withdraw (Lakey & Orehek, 2011). Withdrawal from others can also be a strategy to cope with stigma and shame, as a way of protecting oneself from further harm and stigmatization (Azorina et al., 2019; De Hooge et al., 2010).

The present study confirms other studies that have shown withdrawal to be a prevalent factor in traumatized bereavement (Azorina et al., 2019; Dyregrov, 2003). For example, a study of parents bereaved after sudden and traumatic child death (suicide, sudden infant child death, and accidents), Dyregrov (2003) found self-isolation to be the most influential factor for impaired psychosocial health among the bereaved. Loss of energy was one frequently mentioned explanation for self-isolation and was discussed as an indication of depression, physical illness, or change in lifestyle. Dyregrov (2003) also discussed how personality traits, or feelings of lack of social support can influence on bereaved’ self-isolation. In Azorina and co-workers’ study (2019) on suicide bereavement among young adults, they found bereavement to influence negatively on social relations, both on bereaved’ tendency to self-isolation, and their lack of social confidence. Particularly, participants in this study isolated themselves from partners and close friends. Stigma related to the cause of death was an important explanation for why strains in social networks occurred. Also, the study showed how stigma was reinforced through a reciprocal relationship between self-isolation and others’ withdrawal (Azorina et al., 2019).

As the present study shows that societal stigma accounts for 4% of variations in social support, stigma may not only affect how the support network approaches the bereaved person, but it can also affect how the bereaved person accepts (receives) support. For example, in interviews with parents bereaved by DRD in the END-project, Titlestad et al. (2020) found that the parents did not experience reduced social support from their informal network (e.g. family, friends, and colleagues) but some of them described the support as difficult to accept because it conflicted with their own, internalized and self-imposed stigma. Another study, also from the END-project demonstrated how siblings had learned to be the strong ones in the family during their siblings’ problematic use of drugs, and had unlearned the ability to accept support from others after the death (Dyregrov et al., 2022).

Self-blame did not make any unique significant contribution to variations in social support in the present study but may be an intermediary variable. For example, bereaved persons that experience societal stigma, and blame themselves for the death see e.g. Bottomley et al. (2023), Titlestad et al. (2021a), may end up signalling to their network that they are not worthy of support. Consequently, the bereaved can self-isolate without giving their network a chance to mobilize around them, or the network becomes unsure of what to do because of the signals received, and so hold back from offering support. The assumption of self-blame as an intermediary variable can be supported by De Hooge et al. (2018)’ general study on shame and guilt and its association with social relations, which found guilt (self-blame) to motivate for withdrawal, while shame motivated more for a social approach among the study’ participants. The present study did not include shame as a variable in the regression analysis, and further research is needed to examine whether the same mechanisms can be relevant for DRD bereavement.

## Limitations

There are some limitations to the study’s design that need to be taken into consideration. The study is based on a cross-sectional design, which means that the study’s data provide a picture of participants’ perceptions of the different questions at one single point in time. The results must be interpreted in this context, as a longitudinal design could have given other results, for example regarding how perceived social support changed over time. A cross-sectional design is advantageous in gathering data from many persons within a short time frame, but it makes it difficult to develop findings in terms of causality. Another limitation is the time delay from data collection to publication. Data for this article was collected six years ago, before the pandemic. The pandemic probably has had a different impact in different contexts, for example on societies’ capacity for mobilizing social networks, and people’s attitudes and mindsets. Still, it is reasonable to assume that the mechanisms regarding DRD, stigma, and social support are deeply anchored and institutionalized in society, and hence results of this study will also be relevant for DRD bereaved persons today. For example, self-isolation is one main finding from the present study, which has been found in studies on traumatic bereavement conducted a long time before the pandemic, see e.g., Dyregrov (2003).

Recruitment of participants to the study followed a pragmatic approach, and no upper time-limits were set concerning the time since death. This contributed to a relatively high level of participants but also to a variance in the time since death between six months to 35 years at the time of the survey, actualizing, for example, questions around recall bias among the participants. Thus, the questionnaires relevant to this study primarily relate to the participants’ present situation, such as their perception of positive social support. Nevertheless, a long time having passed since the death can increase the possibility of confounding variables, meaning that the assumptions derived from the study must be interpreted with some degree of caution.

Some limitations also relate to the composition of the sample. Far more women than men are represented in the study (which is also the case with other studies of bereavement, see e.g., Dyregrov et al. (2003) and Feigelman et al. (2011), and the participants generally belonged to the white middle class in Norway. Also, the average age of the deceased (*M* = 31 years) is lower than the average level of persons who died by overdoses in Norway during the recent years (*M* = 45 years) (Norwegian Institute of Public Health, 2023). It is reasonable to assume that the sample does not reflect the general DRD bereaved population, and we should be cautious in drawing generalized assumptions from these results to other populations and other cultures. Different cultures will have different norms for grieving, stigma towards drug use, and gender roles and expectations will differ in different cultural contexts. For example, norms differ regarding who should be mourned, how to emotionally express grief, or whether mourners should be socially included or excluded (Walter, 2010). Also, most of the bereaved persons in the present study reported having a close relation to the deceased at the time of death. This can have influenced the results of the regression analysis, where the bereaved persons’ self-reported closeness to the deceased at the time of death, was included as a control variable that could contribute to variations in social support.

Two of the variables in the present study are measured by single items: self-blame and own withdrawal. If more complex measurements had been used, we probably could have gained more nuanced results. Still, “I blame myself for the death” and “I have withdrawn from others” are clear questions, with only a limited number of options for interpretations from the participants. The subscale used for the measurement of societal stigma is derived from a larger questionnaire on the “Special Grief Question” (SGQ) and developed specifically for the present study and is not a validated questionnaire on stigma. Thus, it showed that it had good internal consistency and may be used in future research on societal stigma.

Results from the regression analysis that show respondents’ gender was statistically insignificant when it came to the proportion of social support received do not correspond with the results from the t-test that shows a small but significant difference between males and females in total perceived social support. While t-tests use a corrected t-value, this is not the case for regression analysis. This may be one explanation for the inconsistency in the findings regarding respondents’ gender. Another explanation is that since the regression analysis uses listwise deletion of missing values, the number of respondents in the t-test analyses and the regression analyses differ. More research on DRD bereavement and gender variations is therefore needed to examine whether there is a valid and consistent variation in males’ and females’ perceptions of social support.

## Implications and Conclusion

Drug-related death (DRD) is a major public health concern both in Europe and the U.S. and those bereaved by DRD run a high risk of experiencing marginalization and severe health consequences in the aftermath of the death. During the 21st century more attention is given to harm reduction strategies, such as education programs for safer use, enhancing peer support, and naloxone supply and administration (Mercer et al., 2021). However, the effects of such programs are not well documented, and more research is needed to examine the extent to which different measures of harm reduction can contribute to a reduction in DRD (Clark et al., 2014). Probably, DRD prevention must follow along several tracks, in which both early intervention strategies, harm reduction, and legislation, must be used as supplementary means to prevent the horrible consequences of illegal drug use. A thorough consideration of the potential side-effects of the different measures for DRD prevention is also needed. For example, criminalization strategies aimed at preventing people from using drugs can contribute to producing stigma toward those using illegal drugs (Scher et al., 2023). Therefore, possible stigmatizing effects must be carefully considered when preventive strategies are drawn up.

This study has shown that societal stigma and own withdrawal account for 12.5% of the variations in DRD bereaved persons’ perceptions of social support. Subgroup variations in perceptions of social support are generally small, but some results can indicate that being older, being a parent, and being a male are associated with being more satisfied with some aspects of social support. The results implicate that awareness is needed to meet the support needs of younger people, women, and people outside the closest family. However more research is needed that includes different cultural contexts, and people from varied backgrounds to examine whether groups of DRD bereaved persons are more vulnerable to not receiving the support they need. This is important knowledge to target support measures and interventions. Furthermore, this study has shown there is a need to address support measures that could stimulate bereaved persons’ ability to meet with others in the same situation. The results thus indicate that stigma and participants’ own, as well as others’, withdrawal can be a barrier for utilizing informal social support such as peer support groups. It is therefore not enough to initiate peer support groups, but such initiatives need to be followed up by an increased public awareness of showing that persons bereaved after DRD are worthy of support.

Generally, there is a need to build up local support networks in communities and to strengthen the potential of informal support networks for the bereaved persons to have access to good, needs-based support in their closest network. In the same vein, it is important to educate society on grief and bereavement in general, and stigmatized deaths in particular. The results from this study point to societal stigma as a prevalent factor in drug-related death bereavement that is closely intertwined with self-isolation on the part of the bereaved persons. Increasing societal awareness of the potentially devastating consequences that can result from stigmatizing attitudes and implementing measures that facilitate communication and openness about these types of deaths may be important steps in increasing public recognition of DRD and bereavement.

## References

[bibr1-00302228241238907] Arnberg F. K. Hultman C. M. Michel P. O. Lundin T. (2012). Social support moderates posttraumatic stress and general distress after disaster. Journal of Traumatic Stress, 25(6), 721–727. 10.1002/jts.21758 23184348

[bibr2-00302228241238907] Azorina V. Morant N. Nesse H. Stevenson F. Osborn D. King M. Pitman A. (2019). The perceived impact of suicide bereavement on specific interpersonal relationships: A qualitative study of survey data. International Journal of Environmental Research and Public Health, 16(10), 1801. 10.3390/ijerph16101801 31117207 PMC6572476

[bibr3-00302228241238907] Barlow C. A. Schiff J. W. Chugh U. Rawlinson D. Hides E. Leith J. (2010). An evaluation of a suicide bereavement peer support program. Death Studies, 34(10), 915–930. 10.1080/07481181003761435 24482855

[bibr4-00302228241238907] Bartone P. T. Bartone J. V. Violanti J. M. Gileno Z. M. (2019). Peer support services for bereaved survivors: A systematic review. Omega, 80(1), 137–166. 10.1177/0030222817728204 28871835

[bibr5-00302228241238907] Bottomley J. S. Campbell K. W. Titlestad K. B. Feigelman W. Rheingold A. A. (2023). Predictors of stigma, guilt, and shame among adults bereaved by fatal overdose. *OMEGA* - Journal of Death and Dying, Article, 302228231194208. 10.1177/00302228231194208 37553120

[bibr6-00302228241238907] Bottomley J. S. Feigelman W. T. Rheingold A. A. (2022). Exploring the mental health correlates of overdose loss. Stress and Health: Journal of the International Society for the Investigation of Stress, 38(2), 350–363. 10.1002/smi.3092 34448352 PMC11267624

[bibr7-00302228241238907] Burke L. A. Neimeyer R. A. (2013). Prospective risk factors for complicated grief: A review of the empirical literature. In Complicated grief: Scientific foundations for healthcare professionals (pp. 145–161). Routledge. 10.4324/9780203105115-21

[bibr8-00302228241238907] Carstensen L. L. Isaacowitz D. M. Charles S. T. (1999). Taking time seriously: A theory of socioemotional selectivity. American Psychologist, 54(3), 165–181. 10.1037/0003-066x.54.3.165 10199217

[bibr9-00302228241238907] Centers for Disease Control and Prevention . (2023). Drug overdose deaths in the United States, 2001 - 2021 [online]. Available at: https://www.cdc.gov/nchs/products/databriefs/db457.htm

[bibr10-00302228241238907] Christiansen S. G. Reneflot A. Stene-Larsen K. Johan Hauge L. (2020). Parental mortality following the loss of a child to a drug-related death. The European Journal of Public Health, 30(6), 1098–1102. 10.1093/eurpub/ckaa094 32535625

[bibr11-00302228241238907] Clark A. K. Wilder C. M. Winstanley E. L. (2014). A systematic review of community opioid overdose prevention and naloxone distribution programs. Journal of Addiction Medicine, 8(3), 153–163. 10.1097/adm.0000000000000034 24874759

[bibr12-00302228241238907] Cobb S. (1976). Social support as a moderator of life stress. Psychosomatic Medicine, 38(5), 300–314. 10.1097/00006842-197609000-00003 981490

[bibr13-00302228241238907] Cohen J. (1988). Statistical power analysis for the behavioral Sciences. Routledge Academic.

[bibr14-00302228241238907] Cohen S. Wills T. A. (1985). Stress, social support, and the buffering hypothesis. Psychological Bulletin, 98(2), 310–357. 10.1037/0033-2909.98.2.310, https://psycnet.apa.org/doi/10.1037/0033-2909.98.2.310 3901065

[bibr15-00302228241238907] De Hooge I. E. Breugelmans S. M. Wagemans F. M. Zeelenberg M. (2018). The social side of shame: Approach versus withdrawal. Cognition & Emotion, 32(8), 1671–1677. 10.1080/02699931.2017.1422696 29303420

[bibr16-00302228241238907] De Hooge I. E. Zeelenberg M. Breugelmans S. M. (2010). Restore and protect motivations following shame. Cognition & Emotion, 24(1), 111–127. 10.1080/02699930802584466

[bibr17-00302228241238907] Doka K. J. (1999). Disenfranchised grief. Bereavement Care, 18(3), 37–39. 10.1080/02682629908657467

[bibr18-00302228241238907] Dyregrov K. (2003). The loss of a child by suicide, SIDS, and accidents: Consequences, needs and provisions of help. Doctoral dissertation (dr. philos). HEMIL, Faculty of Psychology, University of Bergen.

[bibr19-00302228241238907] Dyregrov K. (2006). Experiences of social networks supporting traumatically bereaved. OMEGA - Journal of Death and Dying, 52(4), 339–358. 10.2190/claa-x2lw-jhqj-t2dm

[bibr20-00302228241238907] Dyregrov K. Berntsen G. Silviken A. (2014). The need for and barriers to professional help-a qualitative study of the bereaved in Sámi areas. Suicidology Online, 5(1), 47–58.

[bibr21-00302228241238907] Dyregrov K. Dyregrov A. (2008). Effective grief and bereavement support: The role of family, friends, colleagues, schools and support professionals. Jessica Kingsley Publishers.

[bibr22-00302228241238907] Dyregrov K. Møgster B. Løseth H. M. Lorås L. Titlestad K. B. (2020). The special grief following drug related deaths. Addiction Research and Theory, 28(5), 415–424. 10.1080/16066359.2019.1679122

[bibr23-00302228241238907] Dyregrov K. Nordanger D. Dyregrov A. (2003). Predictors of psychosocial distress after suicide, SIDS and accidents. Death Studies, 27(2), 143–165. 10.1080/07481180302892 12678058

[bibr24-00302228241238907] Dyregrov K. Selseng L. B. (2022). “Nothing to mourn, He was just a drug addict” - stigma towards people bereaved by drug-related death. Addiction Research and Theory, 30(1), 5–15. 10.1080/16066359.2021.1912327

[bibr25-00302228241238907] Dyregrov K. Titlestad K. B. Selseng L. B. (2022). Why informal support fails for siblings bereaved by a drug-related death: A qualitative and interactional perspective. OMEGA - Journal of Death and Dying, Article, 003022282211293. 10.1177/00302228221129372 PMC11776349 36154325

[bibr26-00302228241238907] Elklit A. Kurdahl S. (2013). The psychological reactions after witnessing a killing in public in a Danish high school. European Journal of Psychotraumatology, 4(1), Article 19826. 10.3402/ejpt.v4i0.19826 PMC3542399 23316270

[bibr27-00302228241238907] Elklit A. Schmidt Pedersen S. Jind L. (2001). The crisis support scale: Psychometric qualities and further validation. Personality and Individual Differences, 31(8), 1291–1302. 10.1016/s0191-8869(00)00220-8

[bibr28-00302228241238907] Elklit A. O’Connor M. (2005). Post‐traumatic stress disorder in a Danish population of elderly bereaved. Scandinavian Journal of Psychology, 46(5), 439–445. 10.1111/j.1467-9450.2005.00475.x 16179026

[bibr29-00302228241238907] Feigelman W. Feigelman B. Range L. M. (2020). Grief and healing trajectories of drug-death-bereaved parents. Omega, 80(4), 629–647. 10.1177/0030222818754669 29357755

[bibr30-00302228241238907] Feigelman W. Jordan J. R. Gorman B. S. (2011). Parental grief after a child's drug death compared to other death causes: Investigating a greatly neglected bereavement population. Omega, 63(4), 291–316. 10.2190/OM.63.4.a 22010370

[bibr31-00302228241238907] Feigelman W. Jordan J. R. McIntosh J. L. Feigelman B. (2012). Devastating losses: How parents cope with the death of a child to suicide or drugs. Springer Publishing Company.

[bibr32-00302228241238907] Gilbert K. R. (1996). “We’ve had the same loss, why don't we have the same grief?” loss and differential grief in families. Death Studies, 20(3), 269–283. 10.1080/07481189608252781

[bibr33-00302228241238907] Goffman E. (2009). Stigma: Notes on the management of spoiled identity. Simon and Schuster.

[bibr34-00302228241238907] Hibberd R. Elwood L. S. Galovski T. E. (2010). Risk and protective factors for posttraumatic stress disorder, prolonged grief, and depression in survivors of the violent death of a loved one. Journal of Loss & Trauma, 15(5), 426–447. 10.1080/15325024.2010.507660

[bibr35-00302228241238907] Jind L. Elklit A. Christiansen D. (2010). Cognitive schemata and processing among parents bereaved by infant death. Journal of Clinical Psychology in Medical Settings, 17(4), 366–377. 10.1007/s10880-010-9216-1 21110073

[bibr36-00302228241238907] Johnsen I. Dyregrov K. (2016). “Only a Friend” the bereavement process of young adults after the loss of a close friend in an extreme terror incident—a qualitative approach. OMEGA - Journal of Death and Dying, 74(1), 16–34. 10.1177/0030222815622956

[bibr37-00302228241238907] Joseph S. (1999). Social support and mental health following trauma. In Yule W. (Ed.), Post-traumatic stress disorders: Concepts and therapy (pp. 71–91). John Wiley & Sons Ltd.

[bibr38-00302228241238907] Joseph S. Andrews B. Williams R. Yule W. (1992). Crisis support and psychiatric symptomatology in adult survivors of the Jupiter cruise ship disaster. British Journal of Clinical Psychology, 31(1), 63–73. 10.1111/j.2044-8260.1992.tb00968.x 1559118

[bibr39-00302228241238907] Kalsås Ø. R. Dyregrov K. Fadnes L. T. Titlestad K. B. (2023). The social health domain of persons bereaved by a drug-related death and associations with professional help: A cross-sectional study. Death Studies, 47(8), 1–12. 10.1080/07481187.2022.2142329 36347016

[bibr40-00302228241238907] Kheibari A. Cerel J. Victor G. (2021). Comparing attitudes toward stigmatized deaths: Suicide and opioid overdose deaths. International Journal of Mental Health and Addiction, 20(4), 2291–2305. 10.1007/s11469-021-00514-1

[bibr41-00302228241238907] Lakey B. Orehek E. (2011). Relational regulation theory: A new approach to explain the link between perceived social support and mental health. Psychological Review, 118(3), 482–495. 10.1037/a0023477 21534704

[bibr42-00302228241238907] Link B. G. Phelan J. C. (2001). Conceptualizing stigma. Annual Review of Sociology, 27(1), 363–385. 10.1146/annurev.soc.27.1.363

[bibr43-00302228241238907] Liu W.-M. Forbat L. Anderson K. (2019). Death of a close friend: Short and long-term impacts on physical, psychological and social well-being. PLoS One, 14(4), Article e0214838. 10.1371/journal.pone.0214838 30947290 PMC6448887

[bibr44-00302228241238907] Lobb E. A. Kristjanson L. J. Aoun S. M. Monterosso L. Halkett G. K. Davies A. (2010). Predictors of complicated grief: A systematic review of empirical studies. Death Studies, 34(8), 673–698. 10.1080/07481187.2010.496686 24482845

[bibr45-00302228241238907] Logan E. L. Thornton J. A. Breen L. J. (2018). What determines supportive behaviors following bereavement? A systematic review and call to action. Death Studies, 42(2), 104–114. 10.1080/07481187.2017.1329760 28494205

[bibr46-00302228241238907] Matud M. P. Ibañez I. Bethencourt J. M. Marrero R. Carballeira M. (2003). Structural gender differences in perceived social support. Personality and Individual Differences, 35(8), 1919–1929. 10.1016/s0191-8869(03)00041-2

[bibr47-00302228241238907] Mercer F. Miler J. A. Pauly B. Carver H. Hnízdilová K. Foster R. Parkes T. (2021). Peer support and overdose prevention responses: A systematic ‘state-of-the-art’review. International Journal of Environmental Research and Public Health, 18(22), Article 12073. 10.3390/ijerph182212073 34831839 PMC8621858

[bibr48-00302228241238907] Norwegian Institute of Public Health . (2023). Narkotikautløste dødsfall 2022 [Drug-related deaths 2022] [online]. Available at: https://www.fhi.no/le/rusmidler-og-avhengighet/narkotikainorge/konsekvenser-av-narkotikabruk/narkotikautloste-dodsfall/?term=.

[bibr49-00302228241238907] O’Connor M. (2010). PTSD in older bereaved people. Aging & Mental Health, 14(6), 670–678. 10.1080/13607860903311725 20686978

[bibr50-00302228241238907] Peskin H. (2019). Who has the right to mourn? Relational deference and the ranking of grief. Psychoanalytic Dialogues, 29(4), 477–492. 10.1080/10481885.2019.1632655

[bibr51-00302228241238907] Reevy G. M. Maslach C. (2001). Use of social support: Gender and personality differences. Sex Roles, 44(7/8), 437–459. 10.1023/a:1011930128829, https://link.springer.com/article/10.1023/A:1011930128829

[bibr52-00302228241238907] Scher B. D. Neufeld S. D. Butler A. Bonn M. Zakimi N. Farrell J. Greer A. (2023). “Criminalization Causes the Stigma”: Perspectives from people who use drugs. Contemporary Drug Problems, 50(3), 402–425. 10.21428/cb6ab371.607a74c3

[bibr53-00302228241238907] Scott H. R. Pitman A. Kozhuharova P. Lloyd-Evans B. (2020). A systematic review of studies describing the influence of informal social support on psychological wellbeing in persons bereaved by sudden or violent causes of death. BMC Psychiatry, 20(1), 1–20. 10.21203/rs.2.16944/v3 32471407 PMC7257446

[bibr54-00302228241238907] Sheehan L. Corrigan P. (2020). Stigma of disease and its impact on health. In The Wiley Encyclopedia of Health Psychology (pp. 57–65). John Wiley & Sons. 10.1002/9781119057840.ch139

[bibr55-00302228241238907] Stout J. H. (2022). The impact of stigma on family and friends bereaved by a drug overdose death. PhD. University of Delaware. https://udspace.udel.edu/server/api/core/bitstreams/de8a1f73-2eda-4e4f-907b-494680b32903/content

[bibr56-00302228241238907] Stout J. H. Fleury-Steiner B. (2023). Stigmatized bereavement: A qualitative study on the impacts of stigma for those bereaved by a drug-related death. OMEGA - Journal of Death and Dying, Article, 302228231203355. 10.1177/00302228231203355 37725891

[bibr57-00302228241238907] Stroebe M. Stroebe W. Schut H. (2001). Gender differences in adjustment to bereavement: An empirical and theoretical review. Review of General Psychology, 5(1), 62–83. 10.1037/1089-2680.5.1.62

[bibr58-00302228241238907] Taylor S. E. (2011). Social support: A review. In Friedman H. S. (Ed.), The Oxford handbook of health psychology (pp. 189–214). Oxford University Press.

[bibr59-00302228241238907] Titlestad K. B. Dyregrov K. (2022). Does ‘time heal all wounds? ’The prevalence and predictors of prolonged grief among drug-death bereaved family members: A cross-sectional study. OMEGA - Journal of Death and Dying, Article, 003022282210985. 10.1177/00302228221098584 PMC11423554 35482973

[bibr60-00302228241238907] Titlestad K. B. Lindeman S. K. Lund H. Dyregrov K. (2021a). How do family members experience drug death bereavement? A systematic review of the literature. Death Studies, 45(7), 508–521. 10.1080/07481187.2019.1649085 31390307

[bibr61-00302228241238907] Titlestad K. B. Mellingen S. Stroebe M. Dyregrov K. (2021b). Sounds of silence. The “special grief” of drug-death bereaved parents: A qualitative study. Addiction Research and Theory, 29(2), 155–165. 10.1080/16066359.2020.1751827

[bibr62-00302228241238907] Titlestad K. B. Schmid M. T. Dyregrov K. (2022). Prevalence and predictors of prolonged grief symptoms among those bereaved from a drug-related death in a convenience sample of Norwegian parents: A cross-sectional study. Death Studies, 46(6), 1354–1363. 10.1080/07481187.2020.1867255 33427100

[bibr63-00302228241238907] Titlestad K. B. Stroebe M. Dyregrov K. (2020). How do drug-death-bereaved parents adjust to life without the deceased? A qualitative study. Omega, 82(1), 141–164. 10.1177/0030222820923168 32389093 PMC7418270

[bibr64-00302228241238907] Treml J. Linde K. Engel C. Glaesmer H. Hinz A. Luck T. Riedel-Heller S. Sander C. Kersting A. Kersting A. (2022). Loss and grief in elderly people: Results from the LIFE-adult-study. Death Studies, 46(7), 1621–1630. 10.1080/07481187.2020.1824203 32972330

[bibr65-00302228241238907] Valentine C. Bauld L. Walter T. (2016). Bereavement following substance misuse: A disenfranchised grief. OMEGA - Journal of Death and Dying, 72(4), 283–301. 10.1177/0030222815625174

[bibr66-00302228241238907] Vangelisti A. L. (2009). Challenges in conceptualizing social support. Journal of Social and Personal Relationships, 26(1), 39–51. 10.1177/0265407509105520

[bibr67-00302228241238907] Wahlström L. Michélsen H. Schulman A. Backheden M. (2013). Support, opinion of support and psychological health among survivors of a natural disaster. International Journal of Social Psychiatry, 59(1), 40–47. 10.1177/0020764011423174 21971982

[bibr68-00302228241238907] Walter T. (2010). Grief and culture: A checklist. Bereavement Care, 29(2), 5–9. 10.1080/02682621003707431

[bibr69-00302228241238907] Wethington E. Kessler R. C. (1986). Perceived support, received support, and adjustment to stressful life events. Journal of Health and Social behavior, 27(1), 78–89. 10.2307/2136504 3711634

[bibr70-00302228241238907] Wilsey S. A. Shear M. K. (2007). Descriptions of social support in treatment narratives of complicated grievers. Death Studies, 31(9), 801–819. 10.1080/07481180701537261 17886412

[bibr71-00302228241238907] Wrosch C. Rueggeberg R. Hoppmann C. A. (2013). Satisfaction with social support in older adulthood: The influence of social support changes and goal adjustment capacities. Psychology and Aging, 28(3), 875–885. 10.1037/a0032730 23772983

